# Epstein-Barr-virus-specific IgA and IgG serum antibodies in nasopharyngeal carcinoma.

**DOI:** 10.1038/bjc.1976.228

**Published:** 1976-12

**Authors:** H. C. Ho, M. H. Ng, H. C. Kwan, J. C. Chau

## Abstract

The sera of 73 patients with nasopharyngeal carcinoma (NPC), 28 patients with other carcinomas (OC) and 89 healthy subjects (HS) were tested for IgG and IgA antibodies to Epstein-Barr virus (EBV) viral capsid antigen (VCA). The majority of the NPC sera had IgG titres of 160 or above, whereas the majority of the other sera had titres below 160. For IgA reactivity to EBV-VCA, 68 of 73 (93-2%) NPC sera had titres of greater than or equal to 10. In contrast, only 6 of 28 (21-4%) OC sera and none of the HS sera had such titres. The mean serum concentrations of IgG, IgA, IgM and C3' were also determined in 55 NPC and 20 OC patients and 18 HS. They were all significantly higher in the NPC sera than in the HS. Although the concentrations of IgG and C3' were not significantly different in the two carcinoma groups, the concentrations of IgA and IgM were significantly higher in the NPC group than in OC. These findings appear to reflect the intensity of EBV-specific antigenic stimulation in NPC, and the EBV-specific serum IgA reactivity may be a sueful aid to the diagnosis of NPC, especially in cases with an occult primary tumour. It may be also of value as a screening test in people at high risk.


*Br. J. Cancer* (1976) **34**, 655

### EPSTEIN–BARR-VIRUS-SPECIFIC IgA AND IgG SERUM ANTIBODIES IN NASOPHARYNGEAL CARCINOMA

H. C. HO,* MUN H. NG,† H. C. KWAN* AND J. C. W. CHAU*

*From the *Medical and Health Department, Institute of Radiology and Oncology, Queen Elizabeth Hospital, Kowloon, Hong Kong, and the †Department of Microbiology, University of Hong Kong, Hong Kong*

Received 2 July 1976 Accepted 5 August 1976

**Summary.—The sera of 73 patients with nasopharyngeal carcinoma (NPC), 28 patients with other carcinomas (OC) and 89 healthy subjects (HS) were tested for IgG and IgA antibodies to Epstein–Barr virus (EBV) viral capsid antigen (VCA). The majority of the NPC sera had IgG titres of 160 or above, whereas the majority of the other sera had titres below 160. For IgA reactivity to EBV-VCA, 68 of 73 (93·2%) NPC sera had titres of $\geqslant$ 10. In contrast, only 6 of 28 (21·4%) OC sera and none of the HS sera had such titres. The mean serum concentrations of IgG, IgA, IgM and $C_3'$ were also determined in 55 NPC and 20 OC patients and 18 HS. They were all significantly higher in the NPC sera than in the HS. Although the concentrations of IgG and $C_3'$ were not significantly different in the two carcinoma groups, the concentrations of IgA and IgM were significantly higher in the NPC group than in OC. These findings appear to reflect the intensity of EBV-specific antigenic stimulation in NPC, and the EBV-specific serum IgA reactivity may be a useful aid to the diagnosis of NPC, especially in cases with an occult primary tumour. It may be also of value as a screening test in people at high risk.**

An association of the Epstein–Barr virus (EBV) with nasopharyngeal carcinoma (NPC) is now firmly established. Old *et al.* (1966) first demonstrated the presence of precipitating antibodies to EBV-related antigens in sera from patients with the cancer. This discovery was followed by the demonstration that NPC patients in widely separated parts of the world had higher geometric mean titres (GMT) of antibodies to EB viral capsid antigen (VCA) than those of control groups, made up of patients with other head and neck cancers and normal subjects (de Schryver *et al.*, 1969. 1974; Henle *et al.*, 1970; Lin *et al.*, 1971; Henderson *et al.*, 1974; Desgranges *et al.*, 1975). The GMT of antibodies to VCA increases with advancing clinical stage of the disease (Ho, 1970; Henle *et al.*, 1970, 1973; de-The *et al.*, 1975). de-The *et al.* (1975) demonstrated that VCA titre correlated with titres of antibodies to 3 other EBV-specific antigens: early antigen (EA), nuclear antigen (EBNA) and soluble antigen (CF/S). Henle *et al.* (1973) showed in NPC, that antibodies to the diffuse (D) component of the EBV-induced EA was not usually demonstrable in Stage I of the disease, but from Stage II onward there was an increasingly higher titre. Thus, it would seem that the various EBV antibodies are related to the total tumour burden.

Wara *et al.* (1975) reported elevated levels of IgA in NPC patients. Henle and Henle (1976), stimulated by this report, investigated the levels of serum IgA antibodies to VCA and to diffuse (D) or restricted (R) components of the EBV-induced EA complex in NPC patients and control subjects. They found NPC sera to be outstanding in that, prior to specific therapy, 93% of the sera revealed IgA

antibodies to VCA and 70% to D, often at high titres, and that Stages III and IV patients' sera had higher titres than that from patients at Stages I and II. This may be interpreted as related to total tumour burden. Patients examined 2–6 years after therapy, had only low levels of EBV-specific IgA or none at all, except in those with recurrent disease. Less than 5% of 73 patients with other carcinomas and of 76 healthy donors revealed the presence of such antibodies. Like the Henles, we were prompted to study the IgA antibodies to VCA in sera from Chinese NPC patients and control subjects in Hong Kong. In addition, we also studied the serum IgA, IgG, IgM and complement $C_3'$ concentration of some of these patients and healthy subjects.

### MATERIAL AND METHODS

Sera were obtained before specific therapy from 73 NPC patients (12 Stage I, 3 Stage II, 50 Stage III, 6 Stage IV and 2 Stage V disease, according to Ho's stage classification (Ho, 1970)) and 28 patients with other carcinomas (OC) (14 bronchus, 9 head and neck, 2 cervix, 2 urinary bladder and 1 rectum) and from a group of 89 healthy adult subjects (HS) consisting of 27 volunteers, 44 blood donors and 18 patients due for discharge from traumatic wards of Queen Elizabeth Hospital.

Sera were routinely stored in small aliquots at − 70°C until used. IgG anti-VCA titres were determined according to the method of Henle and Henle (1966) and reacting cell smears prepared from Jijoye cell line with diluted serum aliquots and counter-stained with fluorescein-conjugated (FITC) goat anti-human IgG (Dako, Copenhagen). The results were expressed as the reciprocal of the maximum serum dilution giving positive fluorescent staining of the cell smears. To detect IgA anti-VCA, sera were diluted 1 : 10 with PBS, and similarly reacted with the Jijoye cell smears, which were then counter-stained with FITC goat anti-human IgA (Dako, Copenhagen).

Serum immunoglobulin and complement $C_3'$ concentrations were estimated in some of the sera by the radial immuno-diffusion method (Mancini *et al.*, 1964) using commercial immunoplates (Hyland, U.S.A.). It should be noted that this method may not be wholly quantitative for IgA, which exists in different polymeric forms which interfere with the test.

### RESULTS

The results of the tests for IgG and IgA antibodies to EBV–VCA obtained with the sera from the various groups, are shown in the Fig., and compared in Table I. Of the 73 NPC patients, 68 (93·2%) showed IgA antibodies to VCA at titres of ⩾10. In contrast, the frequency was only 5 of 28 (17·9%) in the OC group and 0% in the HS. The difference in frequency between the NPC and the OC or HS groups is significant, but there is also a significant difference between the OC and HS groups. Of the 5 NPC cases with IgA antibodies to VCA at titres of ⩾10, 4 were among the 12 Stage I and only 1 among the 50 Stage III cases. The correlation between EBV-specific IgA and IgG antibodies is evident from the Fig. ($r$ = 0·81). If an IgG anti-VCA titre of 160 is chosen arbitrarily as the lower limit of the high range, the majority of the NPC sera tested fell

TABLE I.—*IgG and IgA Antibodies to EBV-VCA in Sera of NPC and Control Groups*

| Sera | | IgG anti-VCA | | | IgA anti-VCA | | |
|---|---|---|---|---|---|---|---|
| Source | Number | GMT (95% confidence interval) | Group compared | *P | Number of sera with titre ⩾10 (%) | Group compared | *P |
| NPC | 73 | 750 (914–616) | OC | <0·001 | 68 (93·2) | OC | <0·001 |
| | | | HS | <0·001 | | HS | <0·001 |
| OC | 28 | 149 (190–117) | HS | <0·001 | 5 (17·9) | HS | <0·001 |
| HS | 89 | 58 (70–48) | | | 0 (0) | | |

* Calculated by Student's *t* test.

FIG.—Correlation between IgA and IgG antibody titres to VCA in NPC patients and controls.

above this limit, and the majority of the others below it. Discordance in the correlation was observed in only 6 of 190 sera tested. Two of the 6 were from the OC group and 4 from HS. They all had IgA anti-VCA titre of $<10$ and high IgG anti-VCA titres. None had the reverse combination.

The mean serum concentrations of IgG, IgA, IgM and $C_3'$ for the various groups are shown in Table II. They were significantly higher in the NPC group than in the HS. Although the mean concentrations of IgG and $C_3'$ were not significantly different in the two carcinoma groups, the concentrations of IgA and IgM were significantly higher in the NPC group than in the OC (Table II).

### DISCUSSION

For some time it was thought that, since EBV is lymphotropic, the serological manifestations might not have anything to do with the tumour cells, which are of epithelial origin. Now, EBV–DNA and EBNA have been demonstrated in the anaplastic and poorly differentiated squamous carcinoma cells of fresh NPC biopsies (Wolf, zur Hausen and Becker, 1973; Wolf *et al.*, 1975; Klein *et al.*, 1974; Huang *et al.*, 1974). The carcinoma cells, or at least some of them, have been shown by Glaser *et al.* (1976) to possess the receptor for EBV, and their resident EBV genome could be induced by iododeoxyuridine (IUdR) to express EA. Furthermore Trumper, Epstein and Giovanella (1976) obtained a pure culture of NPC cells by passage of NPC tissue through athymic nude mice, and demonstrated that these cells could be induced by treatment with bromodeoxyuridine (BUdR) to express EA, and produce immature and mature herpesvirus particles which were antigenically related to EBV. The presence of EBV genomes in the tumour cells of NPC is, therefore, beyond doubt. The question is whether they have anything to do with the genesis of NPC.

Henle and Henle (1976) raised the possibility that the IgA antibodies to EBV might originate from the secretory immune system rather than the systemic. Since, as mentioned earlier, the resident EBV genomes in NPC cells could be induced by IUdR to express EA (Glaser, 1976) and by BUdR to express EBV

Table II.—*Mean Serum Protein Concentrations of NPC and Control Groups*

| Sera | | Mean concentrations (mg per 100 ml) ± s.d. | | | | | | | | | | | |
|---|---|---|---|---|---|---|---|---|---|---|---|---|---|
| | | IgG | | | IgA | | | IgM | | | $C_3'$ | | |
| Source | Number | | Group compared | *$P$ | | Group compared | *$P$ | | Group compared | *$P$ | | Group compared | *$P$ |
| NPC | 55 | 1824±621 | OC | †ns | 546±279 | OC | <0·05 | 138±54 | OC | <0·01 | 114±32 | OC | †ns |
| | | | HS | <0·01 | | HS | <0·01 | | HS | <0·01 | | HS | <0·01 |
| OC | 20 | 1536±407 | HS | <0·01 | 390±160 | HS | <0·01 | 84±22 | HS | †ns | 112±24 | HS | <0·01 |
| HS | 18 | 1007±194 | | | 240±51 | | | 93±34 | | | 82±34 | | |

particles (Trumper *et al.*, 1976), it is conceivable that the almost exclusively high frequency and titres of EBV-specific IgA in NPC might be derived locally in response to the tumour. The existence of such a state of antigenic stimulation might be expected to have resulted in a random assortment of serum IgG and IgA anti-VCA. The finding to the contrary therefore appears to indicate that both types of VCA-specific serum immunoglobulins might be produced in concert with one another. The fact that serum IgA anti-VCA was detected largely in NPC patients might then reflect, at least in part, the intensity of antigenic stimulation. Consistent with this interpretation, an overall increase in immunoglobulins and $C_3'$ concentrations was also observed in sera of NPC patients. The present findings, however, do not exclude the occurrence of local stimulation of immune responses by EBV antigens in NPC patients, and this question is being currently assessed directly by measuring EBV-specific antibodies and immunoglobulin levels in the naso-oropharyngeal secretions. Whatever the cause of this serum immunoglobulin manifestation in NPC, the test for EBV-specific serum IgA reactivity appears to be a useful aid to the diagnosis of NPC, especially in those cases with carcinomatous cervical nodal metastases or cranial nerve involvement, but an occult primary tumour. It may also be a useful screening test for the cancer in people of high risk, such as southern Chinese and members of families with multiple cases, who have been shown by Ho (1971, 1972*a*, *b*) to have an increased risk.

We wish to acknowledge with thanks the financial assistance from The Hong Kong Anti-Cancer Society and the International Agency for Research on Cancer (IARC), valuable help from Mr C. M. Lam (Medical Statistician) and helpful advice from Prof. P. Alexander in preparing this paper and Mrs P. Liu for typing this manuscript.

### REFERENCES

DE SCHRYVER, A., FRIBERG, S., JR, KLEIN, G., HENLE, G., HENLE, W., DE-THÉ, G., CLIFFORD, P. & HO, H. C. (1969) Epstein–Barr Virus-associated Antibody Patterns in Carcinoma of the Post-nasal Space. *Clin. exp. Immunol.*, **5**, 443.

DE SCHRYVER, A., KLEIN, G., HENLE, W. & HENLE, G. (1974) EB Virus-associated Antibodies in Caucasian Patients with Carcinoma of the Nasopharynx and in Long-term Survivors after Treatment. *Int. J. Cancer*, **13**, 319.

DESGRANGES, G., WOLF, H., DE-THÉ, G., SHANMUGARATNAM, K., CAMMOUN, N., ELLOUZ, R., KLEIN, G., LENNERT, K., MUNOZ, N. & ZUR HAUSEN, H. (1975) Nasopharyngeal Carcinoma X, Presence of Epstein–Barr Genomes in Separated Epithelial Cells of Tumors in Patients from Singapore, Tunisia and Kenya. *Int. J. Cancer*, **16**, 7.

DE-THÉ G. HO, J. H. C., ABLASHI, D. V., DAY, N. E., MACARIO, A. J. L., MARTIN-BERTHELON, M. CL., PEARSON, G. & SOHIER, R. (1975) Nasopharyngeal Carcinoma IX, Antibodies to EBNA and Correlation with Response to other EBV Antigens in Chinese Patients. *Int. J. Cancer*, **16**, 713.

GLASER, R., DE-THÉ, G., LENOIR, G. & HO, J. H. C. (1976) Superinfection of Epithelial Nasopharyngeal Carcinoma Cells with Epstein–Barr Virus. *Proc. natn. Acad. Sci. U.S.A.*, **73**, 960.

HENDERSON, B. E., LOUIE, E., BOGDANOFF, E., HENLE, W., ALENA, B. & HENLE, G. (1974) Antibodies to Herpes Group Viruses in Patients with Nasopharyngeal and Other Head and Neck Cancers. *Cancer Res.*, **34**, 1207.

HENLE, G. & HENLE, W. (1966) Immunofluorescence in Cells Derived from Burkitt's Lymphoma. *J. Bact.*, **91**, 1248.

HENLE, G. & HENLE, W. (1976) Epstein–Barr Virus-specific IgA Serum Antibodies as an Outstanding Feature of Nasopharyngeal Carcinoma. *Int. J. Cancer*, **17**, 1.

HENLE, W., HENLE, G., HO, H. C., BURTIN, P., CACHIN, Y., CLIFFORD, P., DE SCHRYVER, A., DE-THÉ, G., DIEHL, V. & KLEIN, G. (1970) Antibodies to Epstein–Barr Virus in Nasopharyngeal Carcinoma, Other Head and Neck Neoplasms and Control Groups. *J. natn. Cancer Inst.*, **44**, 225.

HENLE, W., HO, H. C., HENLE, G. & KWAN, H. C. (1973) Antibodies to Epstein–Barr Virus-related Antigen in Nasopharyngeal Carcinomas. Comparison of Active Cases and Long-term Survivors. *J. natn. Cancer Inst.*, **51**, 361.

HO, H. C. (1970) The Natural History and Treatment of Nasopharyngeal Carcinoma (NPC). In *Oncology* 1970. Ed. R. Lee Clark, *et al.* Chicago: Year Book Medical Publishers. p. 1.

HO, J. H. C. (1971) Genetic and Environmental Factors in Nasopharyngeal Carcinoma. In *Recent Advances in Human Tumor Virology and Immunology*. Ed. W. Nakahara *et al.* Tokyo: University of Tokyo Press. p. 275.

HO, J. H. C. (1972*a*) Nasopharyngeal Carcinoma (NPC). *Adv. Cancer Res.*, **15**, 57.

HO, J. H. C. (1972*b*) Current Knowledge of the Epidemiology of Nasopharyngeal Carcinoma—A

Review. In *Oncogenesis and Herpesvirus*. Ed. P. M. Biggs, G. de Thé and L. N. Payne. Lyon: IARC Scientific Publication, **2**, 357.

HUANG, D., HO, J. H. C., HENLE, W. & HENLE, G. (1974) Demonstration of Epstein–Barr Virus-associated Nuclear Antigen in Nasopharyngeal Carcinoma Cells from Fresh Biopsies. *Int. J. Cancer*, **14**, 580.

KLEIN, G., GIOVANELLA, B., LINDAHL, T., FIALKOW, P. J., SINGH, S. & STEHLIN, J. (1974) Direct Evidence for the Presence of Epstein–Barr Virus DNA and Nuclear Antigen in Malignant Epithelial Cells from Patients with Anaplastic Carcinoma of the Nasopharynx. *Proc. natn. Acad. Sci., U.S.A.*, **71**, 4737.

LIN, T. M., YANG, C. S., HO, S. W., CHIOU, J. F., WANG, C. H., TU, S. M., CHEN, K. P., ITO, Y., KAWAMURA, A., JR & HIRAYAMA, T. (1971) Antibodies to Herpes Type Virus in Nasopharyngeal Carcinoma and Control Groups in Taiwan. In *Recent advances in human tumor virology and immunology*. Ed. W. Nakahara *et al.* Tokyo: University of Tokyo Press. p. 309.

MANCINI, G., VEERMAN, J. P., CARBONERA, A. D. & HEREMANS, J. F. (1964) A Single Radial Diffusion Method for the Immunological Quantitation of Proteins. In *Polypeptides of biological fluids, Proc. 11th Colloq.* Ed. H. Paeters. Amsterdam: Elsevier Publishing Co. p. 370.

OLD, L. J., BOYSE, E. A., OETTGEN, H. F., DE HARVEN, E., CLEERING, G., WILLIAMSON, B. & CLIFFORD, P. (1966) Precipitating Antibody in Human Serum to an Antigen Present in Cultured Burkitt's Lymphoma Cells. *Proc. natn. Acad. Sci., U.S.A.*, **56**, 1699.

TRUMPER, P. A., EPSTEIN, M. A. & GIOVANELLA, B. C. (1976) Epstein–Barr Virus and Nasopharyngeal Carcinoma. *Lancet*, i, 686.

WARA, W. M., WARA, D. W., PHILLIPS, T. L. & AMMANN, A. J. (1975) Elevated IgA in Carcinoma of the Nasopharynx. *Cancer, N.Y.*, **35**, 1313.

WOLF, H., ZUR HAUSEN, H. & BECKER, V. (1973) EB Viral Genomes in Epithelial Nasopharyngeal Carcinoma Cells. *Nature, New Biol.*, **244**, 245.

WOLF, H., ZUR HAUSEN, H., KLEIN, G., BECKER, V., HENLE, G. & HENLE, W. (1975) Attempts to Detect Virus-specific DNA Sequences in Human Tumors. III. Epstein–Barr Viral DNA in Non-lymphoid Nasopharyngeal Carcinoma Cells. *Med. Microbiol. Immunol.*, **161**, 15.


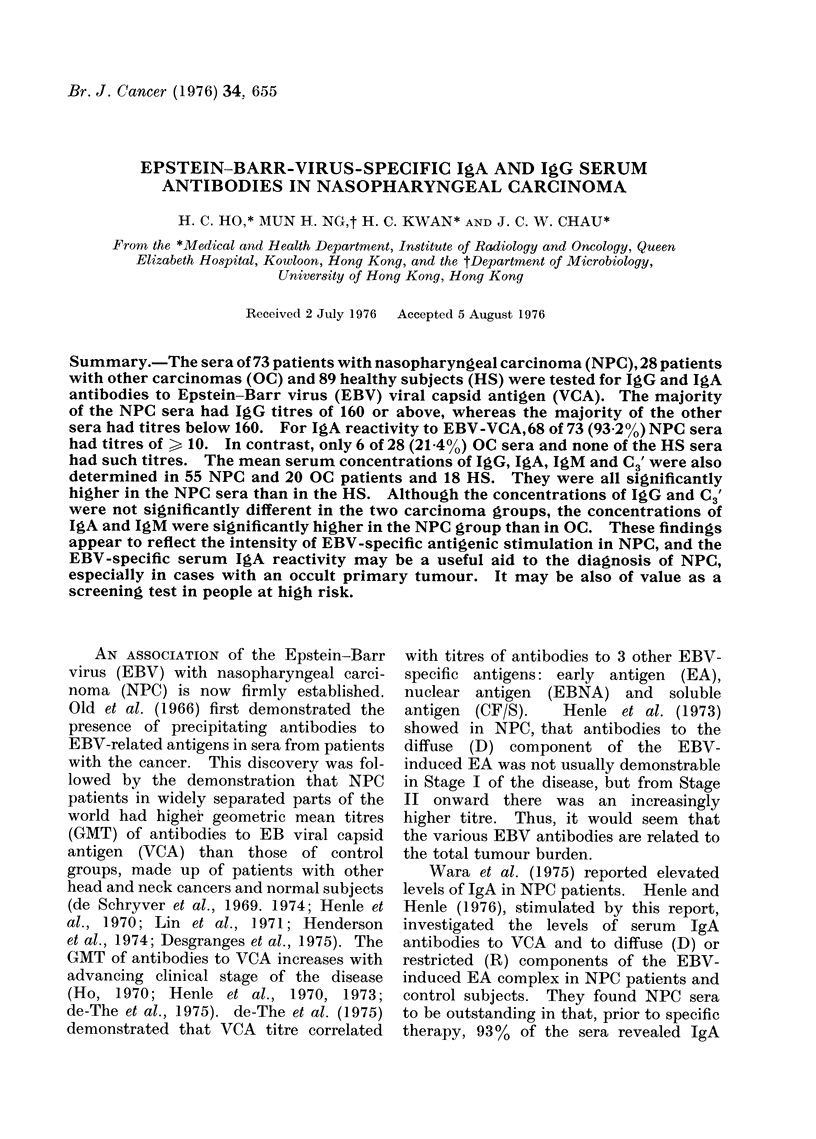

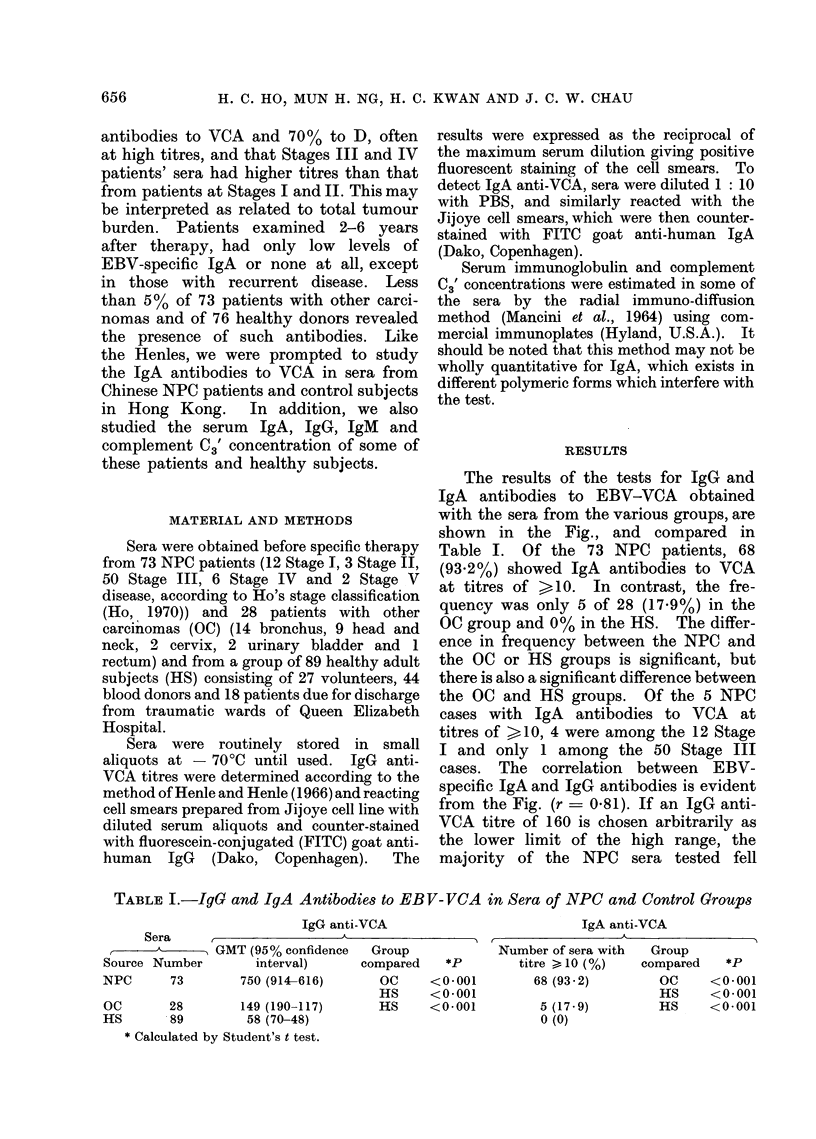

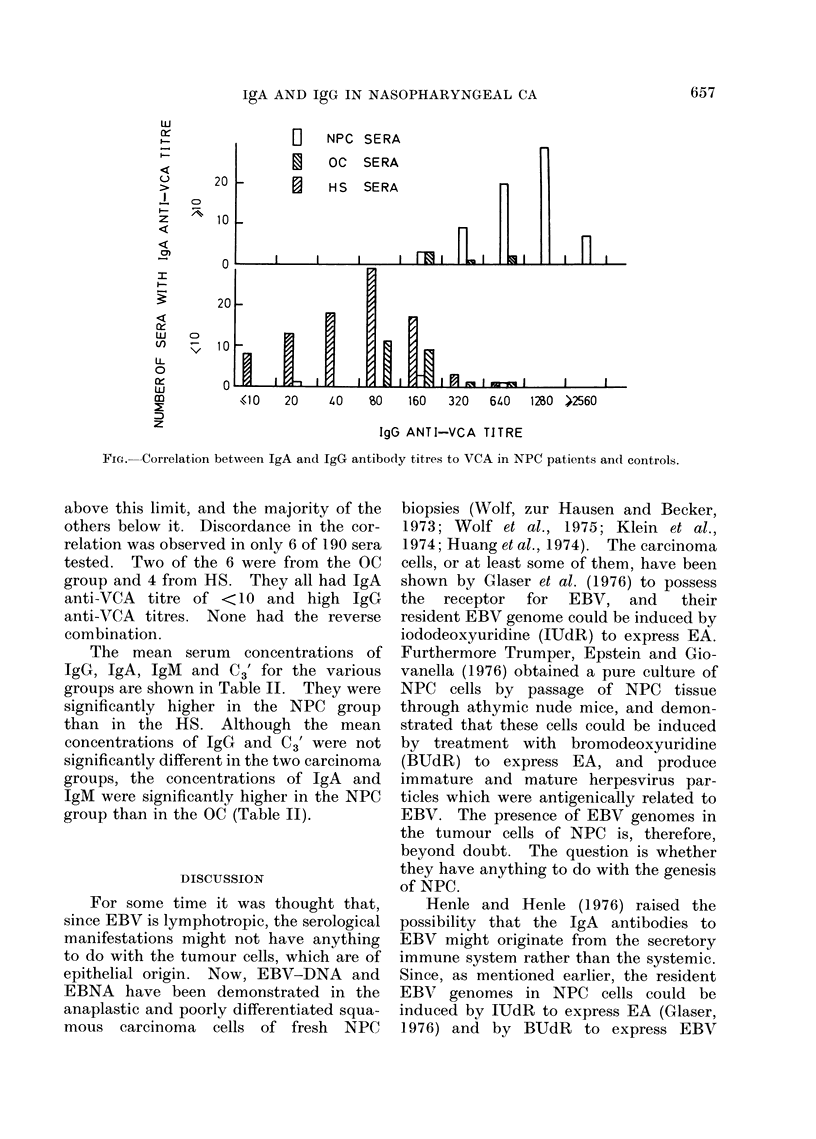

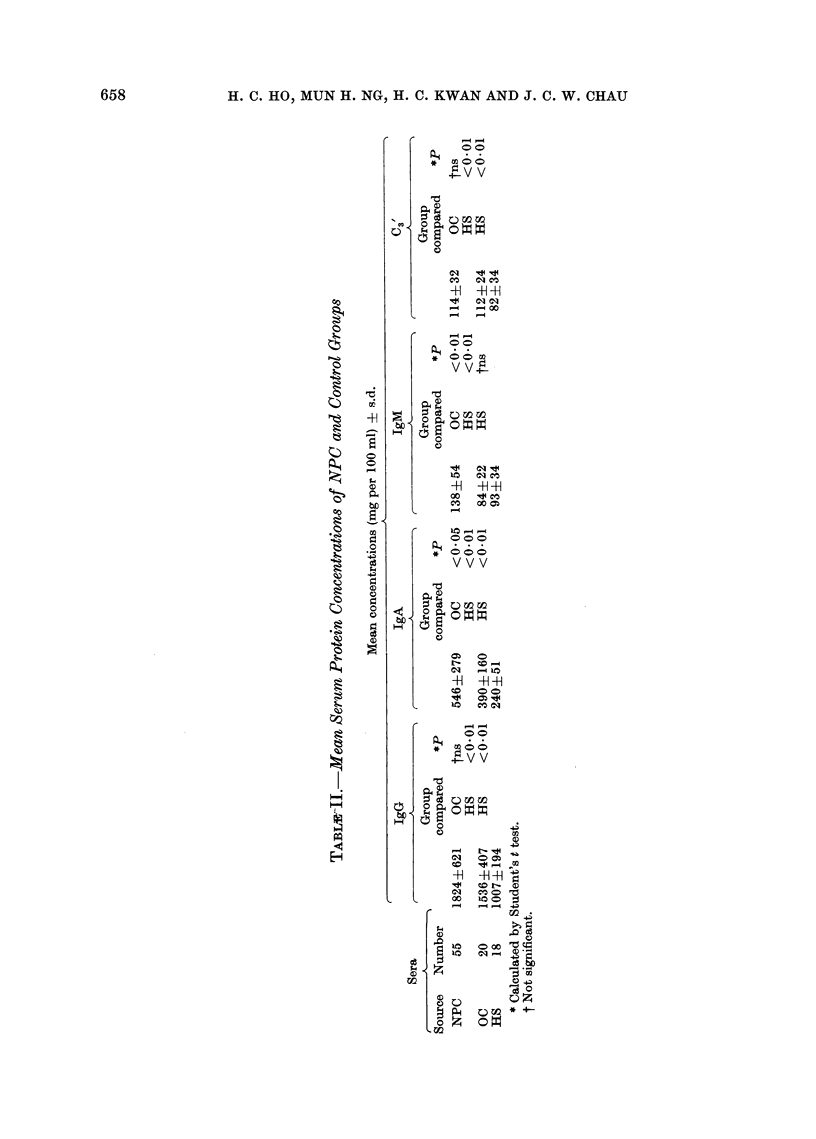

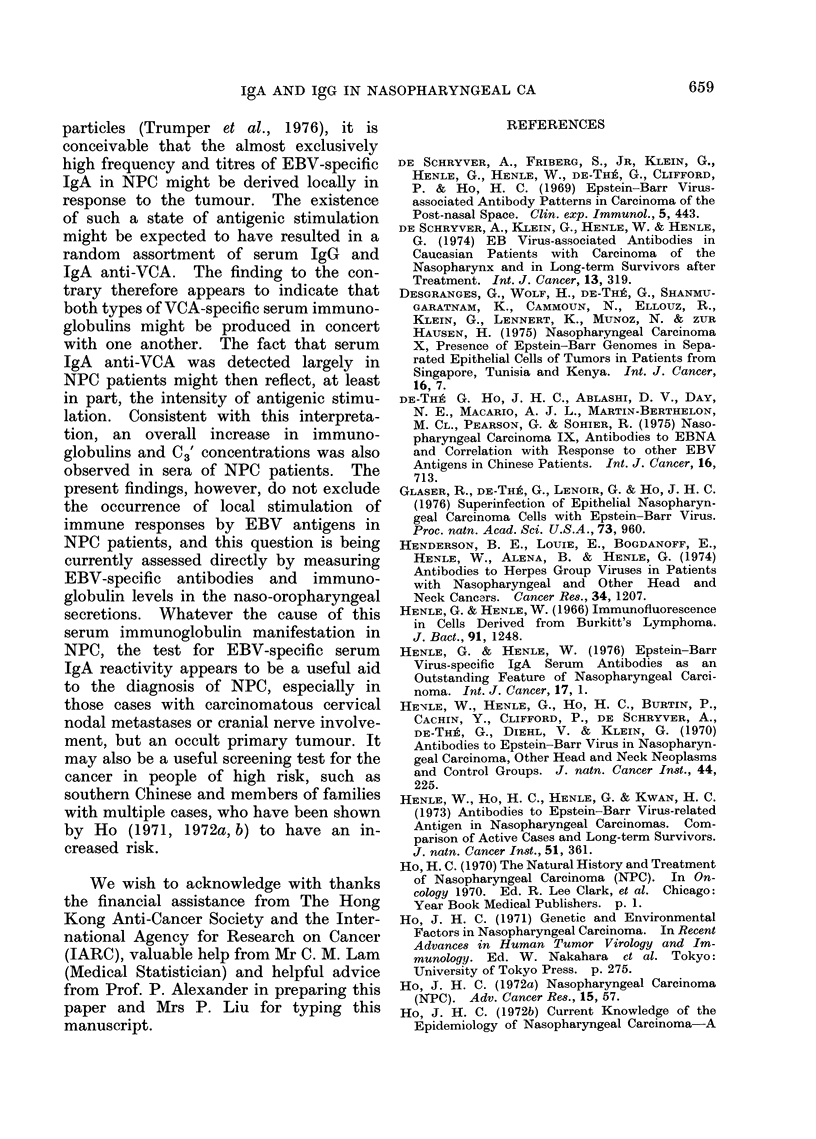

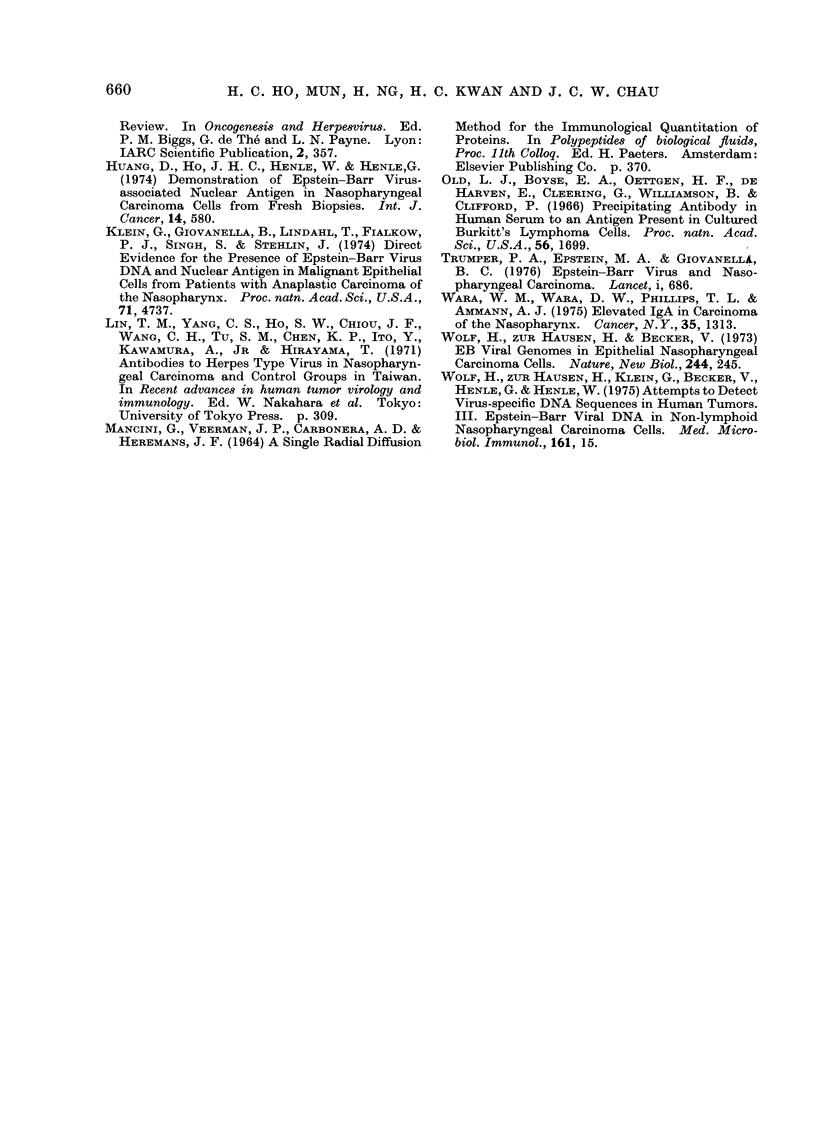


## References

[OCR_00549] Desgranges C., Wolf H., De-Thé G., Shanmugaratnam K., Cammoun N., Ellouz R., Klein G., Lennert K., Muñoz N., Zur Hausen H. (1975). Nasopharyngeal carcinoma. X. Presence of epstein-barr genomes in separated epithelial cells of tumours in patients from Singapore, Tunisia and Kenya.. Int J Cancer.

[OCR_00566] Glaser R., de Thé G., Lenoir G., Ho J. H. (1976). Superinfection epithelial nasopharyngeal carcinoma cells with Epstein-Barr virus.. Proc Natl Acad Sci U S A.

[OCR_00572] Henderson B. E., Louie E., Bogdanoff E., Henie W., Alena B., Henie G. (1974). Antibodies to herpes group viruses in patients with nasopharyngeal and other head and neck cancers.. Cancer Res.

[OCR_00584] Henle G., Henle W. (1976). Epstein-Barr virus-specific IgA serum antibodies as an outstanding feature of nasopharyngeal carcinoma.. Int J Cancer.

[OCR_00579] Henle G., Henle W. (1966). Immunofluorescence in cells derived from Burkitt's lymphoma.. J Bacteriol.

[OCR_00590] Henle W., Henle G., Ho H. C., Burtin P., Cachin Y., Clifford P., de Schryver A., de-Thé G., Diehl V., Klein G. (1970). Antibodies to Epstein-Barr virus in nasopharyngeal carcinoma, other head and neck neoplasms, and control groups.. J Natl Cancer Inst.

[OCR_00599] Henle W., Ho H. C., Henle G., Kwan H. C. (1973). Antibodies to Epstein-Barr virus-related antigens in nasopharyngeal carcinoma. Comparison of active cases with long-term survivors.. J Natl Cancer Inst.

[OCR_00619] Ho J. H. (1972). Nasopharyngeal carcinoma (NPC).. Adv Cancer Res.

[OCR_00633] Huang D. P., Ho J. H., Henle W., Henle G. (1974). Demonstration of Epstein-Barr virus-associated nuclear antigen in nasopharyngeal carcinoma cells from fresh biopsies.. Int J Cancer.

[OCR_00640] Klein G., Giovanella B. C., Lindahl T., Fialkow P. J., Singh S., Stehlin J. S. (1974). Direct evidence for the presence of Epstein-Barr virus DNA and nuclear antigen in malignant epithelial cells from patients with poorly differentiated carcinoma of the nasopharynx.. Proc Natl Acad Sci U S A.

[OCR_00668] Old L. J., Boyse E. A., Oettgen H. F., Harven E. D., Geering G., Williamson B., Clifford P. (1966). Precipitating antibody in human serum to an antigen present in cultured burkitt's lymphoma cells.. Proc Natl Acad Sci U S A.

[OCR_00676] Trumper P. A., Epstein M. A., Giovanella V. S. (1976). Epstein Barr virus and nasopharyngeal carcinoma.. Lancet.

[OCR_00681] Wara W. M., Wara D. W., Phillips T. L., Ammann A. J. (1975). Elevated IGA in carcinoma of the nasopharynx.. Cancer.

[OCR_00691] Wolf H., Zur Hausen H., Klein G., Becker V., Henle G., Henle W. (1975). Attempts to detect virus-specific DNA sequences in human tumors. III. Epstein-Barr viral DNA in non-lymphoid nasopharyngeal carcinoma cells.. Med Microbiol Immunol.

[OCR_00686] Wolf H., zur Hausen H., Becker V. (1973). EB viral genomes in epithelial nasopharyngeal carcinoma cells.. Nat New Biol.

[OCR_00533] de Schryver A., Friberg S., Klein G., Henle W., Henle G., De-Thé G., Clifford P., Ho H. C. (1969). Epstein-Barr virus-associated antibody patterns in carcinoma of the post-nasal space.. Clin Exp Immunol.

[OCR_00540] de Schryver A., Klein G., Henle W., Henle G. (1974). EB virus-associated antibodies in Caucasian patients with carcinoma of the nasopharynx and in long-term survivors after treatment.. Int J Cancer.

[OCR_00557] de-Thé G., Ho J. H., Ablashi D. V., Day N. E., Macario A. J., Martin-Berthelon M. C., Pearson G., Sohier R. (1975). Nasopharyngeal carcinoma. IX. Antibodies to EBNA and correlation with response to other ebv antigens in chinese patients.. Int J Cancer.

